# Low circulating B cells in immunocompromised individuals are linked to poorer antibody responses to vaccines and a predisposition to viral infections

**DOI:** 10.1016/j.jacig.2022.07.008

**Published:** 2022-09-22

**Authors:** Alexandros Grammatikos, Fiona Moghaddas, Henry Reeve, Sarah Johnston, Mark Gompels, Mahableshwar Albur

**Affiliations:** a Bristol Immunology and Allergy Centre, Southmead Hospital, North Bristol NHS Trust, Bristol, United Kingdom; b School of Mathematics, University of Bristol, Bristol, United Kingdom; c Bristol Centre for Antimicrobial Research and Evaluation, North Bristol NHS Trust, Bristol, United Kingdom

**Keywords:** Circulating B cells, viral infections, virus, antibody deficiency, immunodeficiency, viremia, peripheral B cell counts, immunocompromised, immune deficiency, vaccines

## Abstract

**Background:**

B cells play an important role in protection against viral infections, not only through the production of antibodies but also through their ability to act as antigen-presenting cells and produce cytokines.

**Objectives:**

To assess whether there is a link between low circulating B-cell counts and a predisposition to viral infections in immunocompromised individuals, we performed a retrospective cohort analysis at 2 National Health Service Clinical Immunology sites in England.

**Methods:**

Eligible patients were adults who were either diagnosed with or under investigation for an immunodeficiency and had recorded circulating B-cell counts. Information on viral infections was collected by using the departmental, hospital, and laboratory electronic information systems. A generalized linear model was used to analyze the relationship between B-cell counts and relevant indices of viral infection while controlling for patient age, diagnosis group, and T-cell and natural killer cell counts.

**Results:**

A total of 376 eligible patients were identified, 134 of whom had B-cell counts that were below the laboratory-defined refence range (<0.11 ×10^9^/L). Patients with low numbers of circulating B cells had lower pretreatment immunoglobulin levels and poorer antibody responses to vaccines (*Streptococcus pneumonia*, *Clostridium tetani,* and *Haemophilus influenzae type B*). An increased number of chronic or recurrent (*P* = .001), severe or unusual (*P* = .001), and PCR-confirmed viral infections (*P* = .04) were recorded in these patients versus in those with normal numbers of circulating B cells.

**Conclusion:**

Overall, there was a statistically significant association between low circulating B-cell counts and the incidence of clinically important viral infections in this patient cohort, even when controlling for relevant covariates. Clinicians caring for patients with immunodeficiency should be vigilant for these types of infections, particularly in patients with low peripheral B-cell counts. A prospective study will be required to confirm these findings.

## Introduction

Antibody deficiencies are the most common type of immunodeficiencies encountered by clinical immunologists. Patients are predisposed to recurrent sinopulmonary infections by encapsulated bacteria, but viral infections were traditionally thought to be of less concern for them. It is, however, well established that humoral immunity plays an important role in antiviral defenses (eg, antibodies neutralize viral particles, preventing entry into human cells, and participate in the elimination of virally infected cells via opsonization and subsequent phagocytosis). This is, for example, highlighted by the use of mAbs to treat certain viral infections, such as severe acute respiratory syndrome coronavirus 2 (SARS-CoV-2).

A few viral infections have been documented to be more prevalent in patients with antibody deficiency (eg, enteroviral meningoencephalitis in patients with X-linked agammaglobulinemia and those previously treated with rituximab^1^^,^^2^ and chronic norovirus in patients with X-linked agammaglobulinemia and certain patients with common variable immunodeficiency).^3^ Also, latent or chronic lower respiratory viral infections seem to be quite common in primary hypogammaglobulinemia.^4^ Patients who have low or absent circulating B-cell counts (CBCC) may be particularly predisposed to these types of infections.^5^ To assess whether there is a link between a low CBCC and a predisposition to viral infections, we conducted a retrospective cohort analysis in 2 National Health Service Clinical Immunology sites in England.

Adult patients who were either diagnosed with or under investigation for an immunodeficiency were included to the study (see the Supplementary Methods in the Online Repository at www.jaci-global.org). Information on all viral infections during the period which patients were under observation at those sites was collected (mean = 82.2 months per patient; range = 6.1-172.5). Viral infections were classified as (1) chronic, if present for more than 6 weeks; (2) recurrent, if recorded on at least 3 separate occasions more than 3 weeks apart; (3) severe, if invasive or resulting in hospital admission; (4) atypical, if detected in otherwise sterile sites (eg, blood, ocular fluid); and (5) other, if not falling into any of the aforementioned categories. The ratio of viral PCR-positive results to total viral PCR assays performed was also recorded using information provided by the regional laboratory electronic system. CBCC measurements were also collected by using the regional laboratory electronic system (mean = 2.1 measurements per patient; range = 1-14). A generalized linear model was used to analyze the relationship between CBCC and each of the relevant responses while controlling for patient age, diagnosis group, and T-cell and natural killer cell counts.

## Results and discussion

In total, 376 eligible patients were identified, and 134 of them (36%) had a CBCC that was below the laboratory-defined reference range (0.11-0.64 × 10^9^/L). The most common diagnoses recorded were secondary antibody deficiency (n = 89), followed by common variable immunodeficiency (n = 84) (see Table E1 in the Online Repository at www.jaci-global.org). In all, 43% of these patients (n = 162) were receiving immunoglobulin replacement therapy (polyvalent IgG in a dose of 400-600 mg/kg per month at 2- to 4- week intervals). A total of 48 patients who were under investigation for an immunodeficiency were also included.

**Fig E1:**
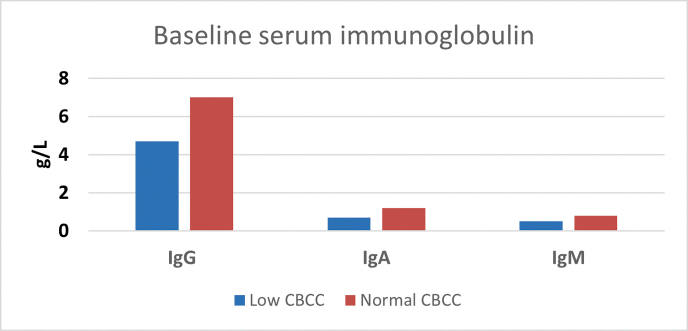


**Fig E2:**
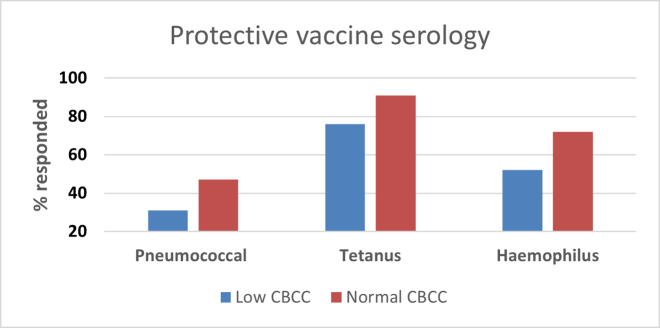


As expected, baseline (pretreatment) immunoglobulin levels were lower in the group of patients with low CBCC (mean IgG = 4.7 g/L; mean IgA = 0.7 g/L, and mean IgM = 0.5 g/L; reference ranges: IgG = 6-16 g/L, IgA = 0.8-4 g/L, IgM = 0.35-2.42 g/L) than in those with normal CBCC (mean IgG = 7 g/L; mean IgA = 1.2 g/L; and mean IgM = 0.8 g/L [see Fig E1 in the Online Repository at www.jaci-global.org]). Patients with low CBCCs also had poorer antibody responses to vaccines (see Fig E2 in the Online Repository at www.jaci-global.org): on average 3.7 out of 12 (31%) *Streptococcus pneumonia* serotypes were positive in low CBCC patients (n = 79), compared with 5.6 of 12 (47%) in those with normal CBCCs (n = 130). *Clostridium tetani* vaccine responses were protective in 13 of 17 patients (76%) with low CBCCs versus in 51 of 56 patients (91%) with normal CBCCs, and *Haemophilus influenzae type B* vaccine responses were protective in 13 of 25 patients (52%) with low CBCCs versus in 42 of 58 patients (72%) with normal CBCCs.

**Fig 1:**
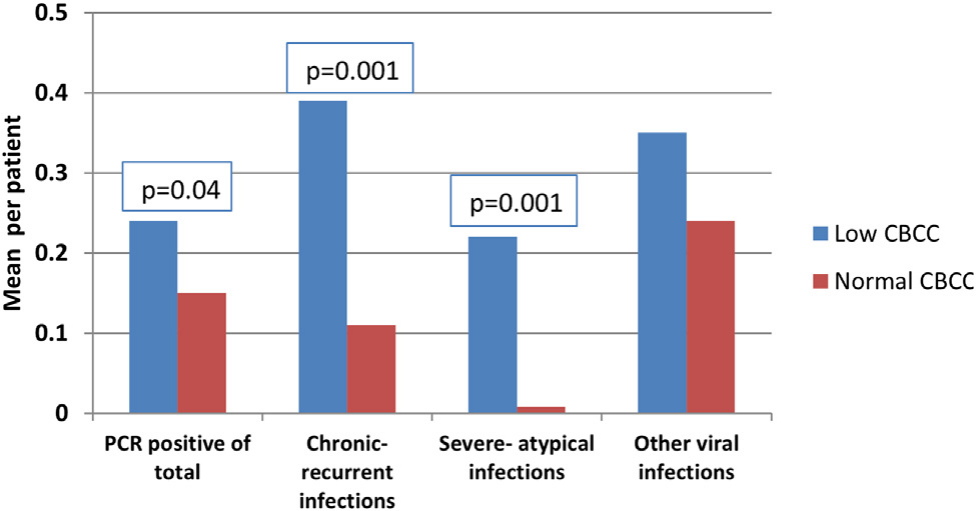
Rate of viral infections in immunocompromised individuals with low and normal CBCCs. The CIs (as a function of B-cell count and additional control variables) were as follows: –4.8 to –0.2 for the ratio of viral PCR-positive results to total viral PCR assays performed, –9.1 to –3.4 for chronic or recurrent viral infections, and –24.1 to –10.2 for severe or atypical viral infections.

Significantly more chronic or recurrent infections were recorded in patients with a low CBCC (mean = 0.39; range = 0-3) than in those with a normal CBCC (mean = 0.11; range 0-1) (*P* = .001; 95% CI = –9.1 to –3.4) (Fig 1). The most common types of infections recorded were cutaneous (herpes simplex [n = 20], warts [n = 12], shingles [n = 8], and molluscum contagiosum [n = 1]), viremia (cytomegalovirus [ n= 8], EBV [n = 7], human herpesvirus 8 [n = 2], human herpesvirus 6 [n = 1], and parvovirus B19 [n = 1]), and intestinal (norovirus [n = 6] and enterovirus [n = 2]) (Tables I and II). Also, significantly more severe or atypical viral infections were recorded in patients with a low CBCC (mean = 0.22; range = 0-3) versus in those with a normal CBCC (mean = 0.008; range = 0-1) (*P* = .001; 95% CI = –24.1 to –10.2) (Fig 1). Finally, significantly more laboratory-confirmed viral infections were recorded in patients with a low CBCC (mean ratio = 0.24) than in those with a normal CBCC (mean ratio = 0.15) (*P* = .04; 95% CI = –4.8 to –0.2) (Fig 1). There was no statistically significant difference in the incidence of other viral infections between patient groups (Fig 1).

**Table I:** Chronic or recurrent and severe or atypical viral infections recorded in the patient cohort (n = 376)

Infection type	No. of patients
Chronic/recurrent	
Cutaneous	41
Viremia	19
Intestinal	8
Urogenital	5
CNS	4
Ocular	2
Respiratory	1
Severe/atypical	
Meningitis/encephalitis	8
Pneumonia	8
Viremia	6
Multidermatomal/disseminated shingles	2
Ocular	2
Lymphoma	1
Post-vaccine infection	1

*CNS*, Central nervous system.

**Table II:** PCR-confirmed viral infections recorded in the patient cohort (n = 376)

Infection type	No. of patients	No. of samples
Respiratory		
Rhinovirus	17	19
Influenza	10	14
Parainfluenza	7	8
SARS-CoV-2	6	20
Adenovirus	6	6
RSV	4	4
CMV	2	2
HSV1	1	1
Metapneumovirus	1	1
Blood		
EBV	12	71
CMV	9	78
Parvovirus B19	2	5
HHV8	2	3
Adenovirus	1	1
HHV6	1	1
Stool		
Norovirus	12	50
Enterovirus	3	5
Cutaneous/oral		
HSV1	8	9
HSV2	4	5
VZV	2	2
CSF		
Enterovirus	3	9
HSV2	2	3
Urogenital		
HSV2	3	4
BK virus	1	11
Ocular		
VZV	2	2

*CMV,* Cytomegalovirus; *HHV,* human herpesvirus; *HSV,* herpes simplex virus; *RSV,* respiratory syncytial virus; *SARS-CoV-2,* severe acute respiratory syndrome coronavirus 2; *VZV,* varicella-zoster virus.

Overall, we found strong evidence of an association between a low CBCC and a clinically significant viral infection risk in immunocompromised individuals while controlling for several relevant cofounders, including patient age, diagnosis group, and T-cell and natural killer cell counts. There are several reasons why a low CBCC may confer such a predisposition. First, B cells directly stimulate cellular immunity and thus activate antiviral defenses. When CBCCs are low, lower IgA and IgM levels are also expected, with poorer IgG antibody responses to infection. Indeed, a significant proportion of patients in our study were receiving immunoglobulin replacement therapy, suggesting that other factors apart from IgG may play a role in preventing viral infections. Consistent with this, a previous study showed that viral infections continue to be problematic in patients with primary antibody deficiency who are receiving immunoglobulin therapy.^6^

The aforementioned findings have important clinical implications. Clinicians caring for patients who harbor immune defects should measure CBCCs and be vigilant for viral infections in patients with low CBCCs. This is of particular importance in the case of severe acute respiratory syndrome coronavirus 2 infection, considering the worse outcomes experienced by immunocompromised patients.^7^ CBCCs may also be used to guide the decision to use live viral vaccines in these patients. According to the current UK guidance, with the exception of oral poliovirus, these vaccines are not contraindicated in antibody-deficient patients.^8^ The US guidance, however, recommends that live viral vaccines be avoided in patients with major antibody deficiencies,^9^ and our findings lend support to this view.

The main strength of our study is its comprehensive nature, with use of extensive clinical and laboratory data to assess the viral infection burden in immunocompromised individuals. Laboratory data alone are unlikely to capture the true burden, as widely available diagnostic tests are lacking for many of these infections. Also, viral infections are interrogated by clinicians less often than bacterial infections. Our findings remained significant with use of 3 different metrics of viral infection, which supports their validity.

Other risk factors, such as sex and certain comorbidities, have been shown to be important in predicting the clinical outcome of viral infections,^10^ but because our study was not designed to investigate this question, such factors were not included in the multivariate analysis. Serum immunoglobulin levels were also excluded because a correlation between pretreatment levels and CBCCs is expected (as seen in Fig E1). Of course, we cannot deduce any causal association based on these observational data alone, and prospective, multicenter studies will be required to confirm the aforementioned findings.

Overall, clinically significant viral infections seem to be more common in immunocompromised individuals with low CBCCs. Physicians caring for these patients should add CBCCs to their diagnostic armamentarium.

**Clinical implications**

Circulating B cells are useful biomarkers to assess the risk of viral infections in immunocompromised individuals.

## Supplementary data

Table E1
