# Supplementary material for: Low circulating B cells in immunocompromised individuals are linked to poorer antibody responses to vaccines and a predisposition to viral infections
Source: J Allergy Clin Immunol Glob. 2022 Sep 22;2(1):111–3. doi: 10.1016/j.jacig.2022.07.008 (PMC10509987; doi:10.1016/j.jacig.2022.07.008)
Supplement: Table E1 [file mmc1.docx]

| **Age (y)** |  |
| --- | --- |
| Mean | 53 |
| Range (minimum) | 19 |
| Range (maximum) | 94 |
| **Sex (n)** |  |
| Male | 226 |
| Female | 150 |
| **Diagnosis (n)** |  |
| Secondary antibody deficiency | 89 |
| CVID | 84 |
| Other primary antibody defects* | 79 |
| Under investigation | 48 |
| Specific antibody deficiency | 32 |
| Combined immunodeficiency | 16 |
| Good’s syndrome | 9 |
| XLA | 6 |
| Immune dysregulation | 5 |
| Complement defects | 4 |
| Other innate immune defects | 4 |

**Suppl. Table I.** Patient characteristics *****other primary antibody defects include IgG subclass deficiency, hyper IgM syndrome, IgA deficiency and unclassified antibody defects (CVID: Common Variable Immunodeficiency, XLA: X-linked agammaglobulinemia).
