# Supplementary material for: Low circulating B cells in immunocompromised individuals are linked to poorer antibody responses to vaccines and a predisposition to viral infections
Source: J Allergy Clin Immunol Glob. 2022 Sep 22;2(1):111–3. doi: 10.1016/j.jacig.2022.07.008 (PMC10509987; doi:10.1016/j.jacig.2022.07.008)
Supplement: Supplementary Methods [file mmc2.docx]

**Supplementary Material**

**Suppl. Methods**

To identify relevant cases, the respective hospital outpatient electronic databases were interrogated. The European Society for Immunodeficiencies diagnostic criteria were applied for diagnosis. HIV positive patients were excluded. Patients that were under investigation for an immunodeficiency at the time of data collection (January to July 2021) were also included to the analysis.

CBCC were measured using Beckton Dickinson FACS Lyric 10-colour flow cytometry and data were obtained by the regional laboratory information system. Patients that did not have recorded B cell levels were excluded from the analysis. For patients with multiple CBCC measurements the most recent result was recorded, except from when this was not relevant to the time-period when viral infections occurred or low CBCC were observed (e.g. patients with previous viraemia or who were on immunosuppressive therapy in the past).

Serum immunoglobulin levels were measured by turbidimetry (Binding Site Optilite). Post vaccine serum antibody titres were determined by an enzyme-linked immunosorbent assay for *Clostridium tetani* and *Haemophilus influenza type B* and by a multiplex assay using bead technology (Bio-Plex 200 by Luminex, Bio-Rad) for *Streptococcus* *pneumonia*. Protective response to vaccination was defined according to laboratory criteria for *Streptococcus* *pneumonia* (≥0.35ug/ml in ≥8 out of 12 serotypes), *Clostridium tetani* (≥0.14 IU/mL) and *Haemophilus influenza type B* (≥0.15mg/L).

Clinical information on viral infections for each patient was collected using the departmental, hospital and laboratory electronic information systems. Free text within clinic letters and relevant correspondence was probed for the terms “*vir*” “wart*”, “HPV”, “shingles”, “chickenpox”, “VZV”, “herpe*”, “HSV”, “EBV”, “CMV”, “glandular”, “COVID*”, and “*flu*”. Hepatitis C viral infections were excluded from the analysis as these tend to become chronic regardless of the immune status of the host.

A generalised linear model was applied to analyse the relationship between B cell count and each of the three variables of interest (PCR positive of total viral infections, chronic or recurrent viral infections, and severe or atypical viral infections), whilst controlling for the patients’ age, diagnosis group, T cell and NK cell count. In particular, Potter’s permutation of regressor residuals test was chosen as this exhibited a clear advantage over the classical Wilk’s likelihood ratio test from the perspective of statistical power, whilst controlling Type 1 error at the nominal level. Confidence intervals and p-values for the coefficient of interest were calculated using R (R Foundation for Statistical Computing, Vienna, Austria).

**Suppl. Figure 1.** Baseline serum immunoglobulin in immunocompromised patients with low and normal CBCC (laboratory defined refence ranges: IgG=6-16g/L, IgA=0.8-4g/L, IgM=0.35-2.42g/L).

**Suppl. Figure 2.** Antibody responses to vaccines in immunocompromised patients with low and normal CBCCs. Left panel: mean percent protective responses to 12 *Streptococcus pneumonia* serotypes tested. Middle & right panels: percent of patients with protective antibody responses to *Clostridium tetani* and *Haemophilus influenza type B* respectively.
